# Okadaic Acid Meet and Greet: An Insight into Detection Methods, Response Strategies and Genotoxic Effects in Marine Invertebrates

**DOI:** 10.3390/md11082829

**Published:** 2013-08-09

**Authors:** María Verónica Prego-Faraldo, Vanessa Valdiglesias, Josefina Méndez, José M. Eirín-López

**Affiliations:** 1 XENOMAR Group, Department of Cellular and Molecular Biology, University of A Coruna, E15071 A Coruña, Spain; E-Mails: veronica.prego@udc.es (M.V.P.-F.); fina@udc.es (J.M.); 2 Toxicology Unit, Department of Psychobiology, University of A Coruña, E15071 A Coruña, Spain; E-Mail: vvaldiglesias@udc.es; 3 Chromatin Structure and Evolution (CHROMEVOL) Group, Department of Biological Sciences, Florida International University, North Miami, FL 33181, USA

**Keywords:** biotoxins, accumulation, depuration, food chain, diarrhetic shellfish poisoning, tumor, apoptosis, genome integrity

## Abstract

Harmful Algal Blooms (HABs) constitute one of the most important sources of contamination in the oceans, producing high concentrations of potentially harmful biotoxins that are accumulated across the food chains. One such biotoxin, Okadaic Acid (OA), is produced by marine dinoflagellates and subsequently accumulated within the tissues of filtering marine organisms feeding on HABs, rapidly spreading to their predators in the food chain and eventually reaching human consumers causing Diarrhetic Shellfish Poisoning (DSP) syndrome. While numerous studies have thoroughly evaluated the effects of OA in mammals, the attention drawn to marine organisms in this regard has been scarce, even though they constitute primary targets for this biotoxin. With this in mind, the present work aimed to provide a timely and comprehensive insight into the current literature on the effect of OA in marine invertebrates, along with the strategies developed by these organisms to respond to its toxic effect together with the most important methods and techniques used for OA detection and evaluation.

## 1. Introduction

Oceans play a seminal role in the different biogeochemical cycles on earth, housing an immense diversity of life forms organized in tightly connected trophic levels throughout different ecosystems. Not surprisingly, such a frail equilibrium is very often disturbed by diverse causes, both natural and anthropogenic. Whichever the origin, the severity of these alterations reach paramount relevance when the natural balance in populations of primary producers, the phytoplankton, is affected. Among the different sources of contamination, massive algal proliferations stand out due to the frequent presence of toxin-producing organisms, constituting Harmful Algal Blooms (HABs). High concentrations of potentially harmful biotoxins are produced and accumulated across the food chains as a result of HABs, causing deleterious effects for organisms in upper trophic levels and threatening the ecosystem integrity [1].

HAB biotoxins are prevalent across European coasts, most notably Diarrhetic Shellfish Poisoning (DSP) toxins are responsible for alterations in the gastrointestinal system of human consumers of contaminated shellfish [2]. Although first documented in Japan [3], the DSP syndrome is now a global disease caused by toxins of the Okadaic Acid (OA) group [4], including OA [5] and its analogs DinophysisToXin-1 (DTX1), dinophysistoxin-2 (DTX2) and their acyl-derivatives, generally known as dinophysitoxin-3 (DTX3). OA constitutes a polyether-type secondary metabolite firstly isolated from the marine sponge *Halichondria okadai* [6] and usually produced by dinoflagellates of the *Dinophysis* and *Prorocentrum* genera [7,8]. Given their lipophilicity, OA toxins are easily accumulated on tissues of filtering marine organisms feeding on HABs, rapidly spreading to their predators in the food chain and eventually reaching human consumers. The negative effects of OA, together with the economic losses associated to HAB episodes, have motivated numerous studies aimed to evaluate the modes of this toxin at cellular and molecular levels. This has been primarily illustrated by research efforts using mammalian cell lines as model systems, revealing the ability of OA to promote tumors and induce apoptosis [9,10]. Nonetheless, the attention drawn to marine organisms in this regard has been scarce so far, even though they constitute primary targets for OA [11].

## 2. Methods Used for the Detection of Okadaic Acid

**Figure 1:**
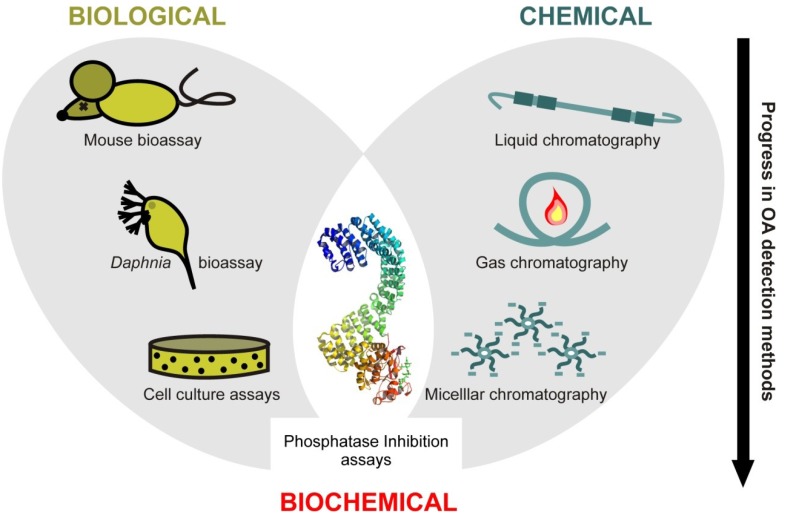
Methods most commonly used for Okadaic Acid (OA) detection and quantification in marine environmental samples.

The great diversity of toxic compounds produced by phytoplankton and their associated bacteria in the sea (marine biotoxins) requires complex detection and quantification strategies. During the last 40 years, the development of such strategies walked hand in hand with the technological progress in life sciences, resulting in a wide range of detection and quantification approaches that can be globally classified into analytical and non-analytical methods, depending on whether or not they are able to unequivocally identify and quantify the toxins in a given sample [12]. Nevertheless, given that different detection methods rely on either biological or chemical (or a combination of both) parameters, the present work addresses them following this classification of biological, chemical and biochemical methods (Figure 1).

### 2.1. Biological Methods

Among the different approaches for detecting marine biotoxins, those based on biological parameters were the first to be developed and are currently the most widely used. The biological detection of OA is based on the study of its toxicological effect on either animals, or tissues or cells. The Mouse BioAssay (MBA) stands out among biological methods because of its wide application [3] and for constituting the standard operating procedure for the detection of OA in food samples (European Union regulation EC No. 2074/2005). Yet, the application of the MBA is hampered by its low specificity and sensitivity as well as relying on the use of test laboratory animals, raising ethical and technical drawbacks [12]. Consequently, the development of alternative methods of improving or replacing the MBA has been fostered by authorities (EC No. 15/2011), including the development of biological detection methods using alternative test organisms such as the planktonic crustacean *Daphnia magna* (Daphnia bioassay), which constitutes an inexpensive tool able to measure OA levels up to 10 times below the threshold of the MBA [13]. Nonetheless, this method still lacks sufficient sensitivity to completely replace the MBA [14]. Similarly, alternative detection strategies based on molecular methodologies have been put forward, including cytotoxic assays based on the study of morphological changes of cultured cell lines exposed to OA [15,16,17]. Such approaches provide increased levels of sensitivity in the detection of OA while abolishing the use of test laboratory animals. Altogether, the progress in the development and optimization of biological methods for OA detection opens up the door to a very promising future of new developments.

### 2.2. Chemical Methods

Although biological methods constitute the preferred approach for detecting marine biotoxins they are unable to provide a quantitative measure of the studied compounds. Such inconvenience has led to the development of chemical detection and quantification methods based on the chromatographic properties of biotoxins [18,19]. The chemical methods most frequently used for the detection of OA are based on Liquid Chromatography (LC) or High Performance Liquid Chromatography (HPLC) separation strategies, coupled with several detection methods including Mass Spectrometry (LC-MS), tandem mass spectrometry (LC-MS/MS), FLuorimetric Detection (HPLC-FLD) and UltraViolet Detection (HPLC-UVD) [12,20,21]. In addition, alternative chromatography-based chemical methods are also available for the detection of OA (though much less used) including Gas Chromatography (GS) [22] and Micellar Electro Kinetic Chromatography (MEKC) [23].

### 2.3. Biochemical Methods

For quite some time, the development of simple, rapid, sensitive, reproductive and inexpensive detection methods for OA has become a major goal, given the critical relevance of this biotoxin during DSP episodes on the European coasts [18,24,25,26]. Within this scenario, the combination of biological and chemical methods has provided the basis for the development of very powerful biochemical strategies currently being applied in the detection and quantification of OA. Among them, the inhibitory effect of this biotoxin on protein phosphatases is the most widely used target in detection routines [27]. This is the case of the Protein Phosphatase 2A (PP2A) inhibition assay, a biochemical method able to accurately detect and quantify OA [28]. Overall, the effectiveness of different methods to detect OA has been widely documented during the last 20 years, with most of them suggesting that both chemical and biochemical strategies could eventually replace the MBA as the standard method for OA detection [29,30,31,32,33,34,35,36] (Figure 1). Nevertheless, the MBA method will still be preferred as long as some biochemical methods keep underestimating the total amount of toxin present in the samples [29]. However, ELISA assays based on direct labeling, which are more sensitive to OA than tests based on indirect labeling, are currently being developed [37].

## 3. Response Strategies to Okadaic Acid in Marine Invertebrates

**Figure 2:**
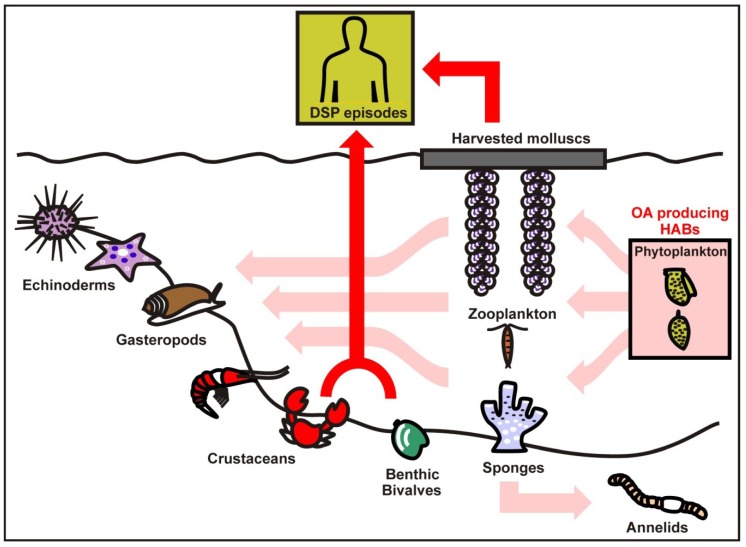
Schematic diagram depicting the transmission of OA across invertebrates in a typical marine food chain. The biotoxin produced by Harmful Algal Blooms (HABs) is initially accumulated by herbivorous consumers including zooplankton, annelids, bivalves and other invertebrates (light pink arrows). OA is subsequently transmitted and further accumulated by their predators, including crustaceans, gasteropods and echinoderms. Bivalves (either harvested or benthic) and crabs (to a lesser extent) are the commonest vectors transmitting OA to human consumers (red arrows) causing Diarrhetic Shellfish Poisoning (DSP) syndrome.

OA encompasses critical relevance in the marine environment of European coasts due to its role in toxic HABs. Furthermore, a progressive increase in OA has also been recently described in North American coasts [38]. Consequently, the study of the mechanisms involved in the accumulation and depuration of OA in marine organisms holds the key for a better understanding of the deleterious effect of this biotoxin in the ecosystems, as well as for the efficient management of toxic episodes, minimizing their effect on human health. So far, the combination of analytical methodologies with the study of sentinel marine organisms has helped understand not only the ways of OA within the cell but also its transmission across the food chain (Figure 2).

### 3.1. Bivalve Molluscs

In bivalve molluscs, OA is mainly absorbed and accumulated in the digestive gland either in a free form or (in the most part) associated with high density soluble lipoproteins [39]. This association results in the sequestration of OA, preventing its transportation to other tissues and hindering its elimination from the organism. On the contrary, free OA is easily transported and quickly removed by means of different passive detoxification mechanisms such as direct OA excretion through the gill or the digestive system [40,41]. In addition, active depuration of OA in bivalves has also been investigated, although it was eventually ruled out by independent studies based on environmental and endogenous factors. On the one hand, it was demonstrated that regulation of OA depuration is insensitive to immediate environmental changes [42]. On the other hand, additional reports indicated that neither organism size nor age play a decisive role in the depuration rate of OA, suggesting that depuration rates cannot be accelerated, even in artificial systems, as a cost-effective way to solve the problem with toxic mussels for the industry [43].

Since most OA is sequestered by lipoproteins in the digestive gland, different studies have proposed that a possible depuration mechanism could involve the association between high density lipoproteins and ATP-Binding Cassette (ABC) transporters, similar to those involved in the removal of excess cholesterol from cells [39,44]. However, what seems now clear is that transformation processes as hydrolysis (during digestion) and, most importantly, acylation are decisive for the metabolism of OA, producing free toxin and esters. Indeed, the rapid bioconversion of OA to 7-*O*-acyl derivates can be taken as a defense mechanism against this biotoxin, though the hydrophobic nature of the esters may slow their elimination from tissues via body fluid [45]. Although a role in OA acylation was initially ascribed to bacteria present in the bivalve gut [46], it was later demonstrated that in mussels the OA is primarily transformed in the endoplasmic reticulum [47]. Acylation seems to play a major role in OA depuration, yet, further studies are still needed in order to ascertain the mechanisms underlying depuration rates of different toxins in different bivalve species [48].

### 3.2. Crabs and Annelids

It is well known that OA is accumulated by different organisms throughout the marine food chains, including mussels, clams, crabs and sponges, among others (Figure 2). Consequently, their predators are also prone to its bioaccumulation, most notably crustaceans, gastropods, starfishes or sea urchins [49]. Indeed, food poisoning episodes caused by OA esters have been reported in human consumers from Portugal and Norway after ingestion of green crab (*Carcinus maenas*) and brown crab (*Cancer pagarus*) [50,51,52], respectively. Not surprisingly, these episodes were coincident with periods of high levels of OA in mussels, although consumption of other bivalves and crustaceans was still permitted. Such scenario supports the study of OA bioaccumulation and depuration also in predators of bivalves, representing a critical objective in improving the safety in the food industry [53].

As for the case of molluscs, annelids are also widely used as sentinel organisms in ecotoxicology studies [54]. Although not directly exposed to OA, different reports have described morphological, functional and toxicological effects in different populations of the annelid *Enchytraeus crypticus*. Firstly, time- and dose-related effects were detected, including swelling of the coelomatic cavity, an increased number of circulating coelomocytes, extension of chloragogenous tissue and general cell suffering in the main animal organs [55]. Secondly, additional studies unveiled an age-dependent effect of OA, with older worms being more sensitive and less able to recover from OA exposure than younger ones [56]. Interestingly, both studies suggested that in this organism the response to OA is coupled with immune response.

### 3.3. Zooplankton and Phytoplankton

Since zooplankton constitutes the trophic level right above phytoplankton, its role as vector of OA in the marine environment is critical and well documented [57,58,59] (Figure 2). However, not all zooplankton species are equally susceptible to biotoxins. For example, the copepods *Temora longicornis* and *Oitona nana*, as well as the tintinnid *Favella serrata*, feed on toxic phytoplankton whereas other copepods such as *Acartia clausi* and *Euterpina acutifrons* do not. Nonetheless, as *T. longicornis* and *O. nana* populations decrease after HABs, the density of *F. serrata* increases thanks to the ingestion of toxic dinoflagellates [58]. These results are supported by previous analysis revealing a co-ocurrence in density peaks of the toxic dinoflagellate *Dinophysis acuminata* and *F. serrata*, suggesting a trophic relationship between both groups [60]. Although some zooplankton species cannot digest toxic phytoplankton they are still able to transfer biotoxins between different trophic levels, as the faecal pellets with undigested dinoflagellates and OA can be assimilated by pelagic coprophagous organisms. In addition, sedimented pellets can be assimilated by a wide community of zooplanktonic organisms (ciliates, harpacticoid copepods, *etc.*) or even by detritivorous bivalves and other benthonic species [58], restarting the cycle all over again.

Besides the known toxic effects of OA on protozoans and metazoans, it has been recently suggested that this biotoxin may also induce cytotoxicity in different algal species, constituting an allelopathic compound against competing microalgae [61,62]. Although the exact mechanism by which OA impairs growth in microalgae is not yet fully known, studies using the green algae *Dunaliella tertiolecta* [63] point towards two major routes: independent and dependent from photosynthesis. Accordingly, when a culture of *D. tertiolecta* is exposed to low OA concentrations, a decrease in cell density is produced both in dark and light conditions, with toxicity being greater in the second case. Additionally, exposure to OA in light conditions results in a reduction of the photosynthetic electron transport rate that may lead to photo-oxidative stress and damage of the photosynthetic apparatus, increasing the observed effect of OA on algal culture cell density.

### 3.4. Role of OA as Defense Mechanism in Marine Organisms

The deleterious effect of OA on different groups of organisms has been depicted throughout this work. Yet, OA can also fulfill useful roles in several organisms primarily as a defense mechanism against pathogens and parasites [64]. This is indeed the case of the marine sponge *Suberites domuncula* harboring bacteria containing OA. Here, low OA concentrations (<100 nM) stimulate the defense system against bacteria [65], whereas high OA concentrations (>500 nM) induce apoptosis in symbiotic or parasitic annelids [65,66] while preventing self-intoxication in the sponge [67,68]. Furthermore, another type of defensive role for OA has been reported in the sponge *Lubomirskia baicalensis*, where OA seems to facilitate the expression of the heat shock protein *hsp70* during the winter season, helping this species withstand water temperatures of 0 °C below the sea ice [69].

## 4. Genotoxic Effects of Okadaic Acid: Lessons from Bivalve Molluscs

Within the cell, OA disrupts the serine/threonine protein phosphatases PP1 and PP2A, leading to a misregulation in the multiple cellular process as well as to cytotoxic and genotoxic effects on the hereditary material. Nonetheless, bivalve molluscs are highly tolerant to OA toxicity [70,71,72], contrasting with the susceptibility displayed by different cell types in other organisms to this biotoxin [17,73,74,75,76]. Different experiments have attempted to study the basis of such tolerance by analyzing the effect of different OA concentrations on mussel blood cells, revealing an increased resistance against sublethal concentrations of this biotoxin as a result of multixenobiotic resistance [72]. These reports conclude that frequent exposure to OA in the marine environment could account for the high tolerance observed in bivalves. In addition, it has been proposed that such resistance could be further increased by OA sequestering in the lysosomal compartment, protecting cells from the cytotoxic effects of this biotoxin.

**Figure 3:**
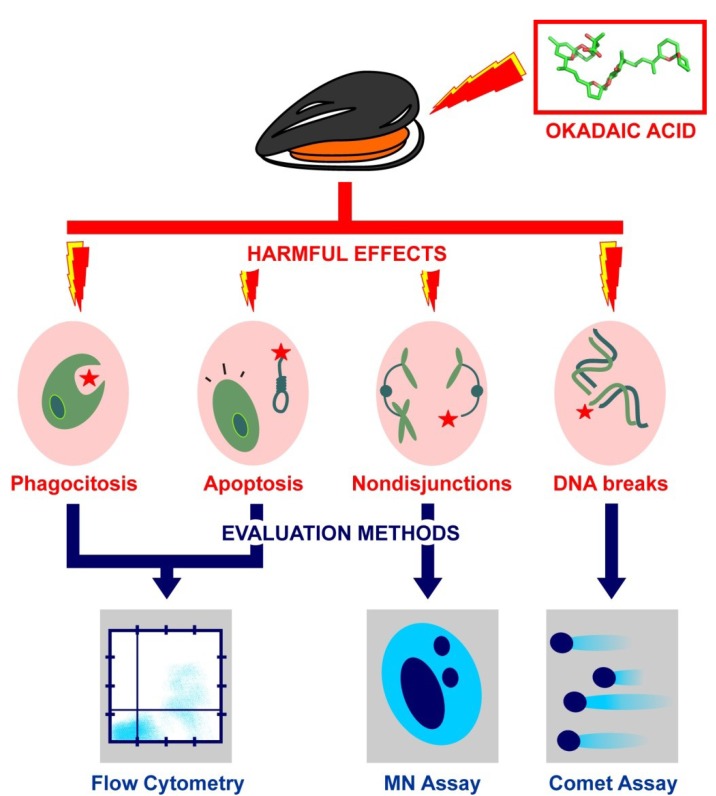
Major genotoxic and cytotoxic effects caused by OA in bivalve molluscs and evaluation methods more frequently used for each specific case.

The molecular mechanisms underlying the deleterious effect of OA have been primarily addressed in mammals [77,78,79,80], revealing a role of this biotoxin in tumorigenesis and induction of apoptosis [81]. On the contrary, the effect of OA in invertebrates is still obscure, with most of the information available referring to bivalve molluscs [70,71,72,82,83,84,85]. In addition to their obvious commercial interest, bivalves constitute preferred organisms for the study of OA given their surprising resistance to this biotoxin [41]. Thus, a growing number of *in vitro* and *in vivo* studies have been carried out during recent years in order to ascertain the genotoxic and cytotoxic effects of OA in bivalve molluscs, especially those encompassing potential applications as pollution biomarkers (Figure 3). Although the development of cDNA microarrays [86] and transcriptomic studies [87] have been very useful for biomonitoring OA genotoxicity using mussels, the most representative experimental approaches are reviewed and discussed below, leaving bioinformatic and Next Generation Sequencing (NGS) technologies for more specialized reports.

### 4.1. Study of OA Effects on Genome Integrity: The Comet Assay

Genome and chromosome integrity are crucial requirements for cell survival that are often jeopardized by the genotoxic effect of OA, most notably by inducing Strand Breaks (SBs) in the DNA [78,88]. However, the level of SBs can serve a very important purpose as an effective genotoxicity biomarker in environmental biomonitoring (Figure 3). The approach most commonly used for the detection and evaluation of DNA SBs is the Single Cell Gel Electrophoresis (SCGE) assay (or simply known as comet assay) which has been recently updated for its application to marine invertebrates [89,90]. More specifically, the comet assay has been used to study the genotoxic effect of OA in the clam *Ruditapes decussatus* [82] by exposing hemocytes to increasing concentrations of OA *in vitro*, revealing a rapid genotoxic effect of OA on the hereditary material. Complementarily, *in vivo* analyses were carried out by feeding clams with different concentrations of the OA-producing dinoflagellate *Prorocentrum lima*. In this case, the study of DNA integrity in hemocytes and gill cells after different exposure periods suggested that OA genotoxicity is dependent upon OA concentration and cell type. Altogether, these data convey critical value for the optimization of the comet assay in bivalves, providing detailed information on the balance between the DNA damage and the activation of repair mechanisms triggered by OA exposure.

### 4.2. Study of OA Effects on Chromosome Integrity: The Micronucleus Assay

In addition to DNA SBs, the effect of OA (through its ability to disrupt PP1 and PP2A phosphatases) often affects chromosomal segregation, increasing the chances of aneuploidies and other cytogenetic abnormalities [91,92,93]. Different *in vitro* cytogenetic assays of chromosomal integrity have been implemented to evaluate OA mutagenicity in somatic cells, most notably the micronucleus assay (Figure 3). This approach, which is based on the detection and quantification of small nuclei carrying chromosome segments resulting from anomalous cell divisions, stands out due to its simplicity and ability to specifically detect chromosomal abnormalities in somatic cells, including chromosome breaks and losses as well as non-disjunction events [72].

The micronucleus assay has been successfully applied for the evaluation of the cytotoxic effects caused by OA in human cell lines, revealing an aneugenic effect which is dependent upon different cell types [80,91,92]. The induction of MicroNuclei (MN) was also corroborated in the case of marine invertebrates using hemocytes of the mussel *Perna perna* [83,84]. In this case, a rapid effect of OA followed by a decrease in MN frequency after a 24 h period was reported, in agreement with previous studies on other bivalve species [94]. Additional analyses corroborated that mussels fed with *Prorocentrum lima* can accumulate enough OA to induce two types of nuclear abnormalities: micronucleus and nucleoplasmic bridges [84]. Again, as in the case of *P. perna*, a significant increase in MN frequency was observed in mussels fed with low concentrations of *P. lima*, whereas MN frequency decreased in mussels subject to high concentrations of this microalgae. Such decrease may be representative of an activation in the repair mechanisms of the cell, counteracting the harmful effects of OA.

### 4.3. Study of OA Effects on Damage Control Mechanisms: Assessment by Flow Cytometry

The harmful effects of OA on the hereditary material constitute potential targets useful for the evaluation of its genotoxic potential. Nonetheless, complementary approaches focused on studying how the molecular machinery of the cell responds to OA have also been developed. This is best illustrated by the characterization of damage control mechanisms involved in the maintenance of genomic integrity. Although studies on such mechanisms are still scarce in marine invertebrates, it has been pointed out that the inhibitory effect of OA on PP1 and/or PP2A phosphatases may be retaliated against by the cell through programmed cell death [95] and/or immune-mediated responses [70,71]. Flow cytometry, a laser-based technique useful for the identification of cells and their components, constitutes the technique of choice for the evaluation of both types of responses in bivalves (Figure 3), unveiling a negative correlation between OA body burden and DNA damage in mussels contaminated with DSP toxins [70]. The study of OA effect was further extended to cell viability, enzymatic status and immune capacity by measuring apoptosis/cell death, non-specific esterase activity and phagocytosis in hemocytes of the clam *Ruditapes decussatus*, respectively. So far, *in vitro* results revealed an increase in apoptosis and cell death as well as a decrease in phagocytosis and esterase activity. In contrast, *in vivo* studies displayed an increase in cell death and esterase activity, together with a dose-independent increase in apoptosis [71].

## 5. Conclusions

Ever since the first documentation on the DSP syndrome in the late 1970s, the interest in the toxins of the OA group has experienced continuous growth fueled by the negative effects of these biotoxins on marine organisms and human consumers, as well as by the economic losses associated to HAB episodes. However, experimental methodologies have not yet reached a unified standard approach able to provide enough sensitivity for the efficient detection of OA, thus hindering the study of the genotoxic effect and response of marine invertebrates to this biotoxin. So far, more than three decades of research in this regard have unveiled that, besides affecting bivalves, OA is also extensively accumulated at all levels of the food chain including by many other edible organisms. Such diversity of OA vectors opens up the door for the future development of biomonitoring programs using these organisms, complementing pre-existing studies based on bivalve molluscs. While this certainly constitutes an attractive objective with relevance for environmental health sciences, further studies will be required in order to improve the detection of OA and tackle its genotoxic effect at the molecular level.
